# Comparison of the Serodiagnostic Accuracy Tests for Lyme Disease in Adults and Children: A Network Meta-Analysis

**DOI:** 10.3390/pathogens14080784

**Published:** 2025-08-06

**Authors:** Weijiang Ma, Jing Li, Li Gao, Xinya Wu, Weijie Ma, Jiaru Yang, Lei Zhong, Jieqin Song, Li Peng, Fukai Bao, Aihua Liu

**Affiliations:** 1 Yunnan Province Key Laboratory of Children’s Major Diseases Research, Department of Pathogens Biology and Immunology, Faculty of Basic Medicine Sciences, Kunming Medical University, Kunming 650500, China; 20221745@kmmu.edu.cn (W.M.); 15082590871@163.com (J.L.); gaoli18469146422@163.com (L.G.); xinyawu1@163.com (X.W.); 13385177456@163.com (W.M.); yjrakm@126.com (J.Y.); zhonglei202202@163.com (L.Z.); jieqinsong@163.com (J.S.); pengli@kmmu.edu.cn (L.P.); 2 Medical Records Room, Suining Municipal Hospital of TCM, Suining 629000, China; 3 Department of Microbiology and Immunology, Haiyuan College of Kunming Medical University, Kunming 650500, China

**Keywords:** Lyme disease, *Borrelia burgdorferi*, serodiagnosis, network meta-analysis, diagnostic test accuracy

## Abstract

As direct detection methods of *Borrelia burgdorferi* are limited, serology plays an important role in diagnosing Lyme disease (LD). There are various types of Lyme serological tests with varying diagnostic accuracy, so it is necessary to compare and rank them. The aim of this study is to compare the accuracy of various serological diagnostic methods for LD using network meta-analysis (NMA). We searched the Cochrane Library and PubMed databases for all serological diagnostic accuracy studies published from the discovery of LD until June 2024. After screening, we assessed the quality of the included studies with QUADAS-C and extracted relevant data. We calculated the Q* index of the receiver operating characteristic curve for each diagnostic test. Meta-disc 2.0 and Stata 15.0 were used to perform traditional meta-analysis and NMA with the gold standard (the comprehensive evaluation) as a reference. We then compared the Q* index values between different methods using two-by-two comparisons and ranked them accordingly. A total of 52 studies with 181,032 participants, including 5318 patients with LD, were included. These studies covered 14 diagnostic methods. The results of the NMA suggest that modified two-tiered testing (MTTT), C6 enzyme immunoassay (EIA), and standard two-tiered testing (STTT) rank in the top three among the 14 methods in terms of Q* index, with MTTT being the highest, followed by C6 EIA and STTT. MTTT and C6 EIA have higher overall diagnostic performance, and their accuracy is not inferior to that of the widely used STTT (PROSPERO CRD42022378326).

## 1. Introduction

Ticks carrying *Borrelia burgdorferi sensu lato* (Bb) can cause Lyme disease (LD), which is a zoonotic infectious disease [1]. LD has wide worldwide prevalence, with cases reported in over 70 countries or regions. According to statistics from the Centers for Disease Control and Prevention (CDC), approximately 476,000 people in the United States receive treatment for LD infections annually [2]. LD can affect multiple systems throughout the body and, based on the progression of the disease, can be categorized into early localized, early disseminated, and late disseminated stages. Erythema migrans (EM) is the early localized first-stage lesion, which alone is sufficient for clinical diagnosis and is present in 75% of cases. EM, Lyme neuroborreliosis (LNB), Lyme carditis, and Lyme arthritis are the most common manifestations of LD [3]. Additionally, other conditions such as acrodermatitis chronica atrophicans (ACA), a late cutaneous manifestation caused by *Borrelia afzelii* and lymphocytoma (early disseminated phase) are also associated with the disease.

The outer membrane of Bb contains a substantial amount of lipoproteins. The vast majority of these proteins can serve as antigens, triggering both innate and adaptive immune responses that subsequently lead to the production of corresponding antibodies. The human immune response to Bb can produce immunoglobulins (Igs); IgM and IgG are the most valuable for the diagnosis of LD [4]. Correct and effective diagnosis is of great significance for optimal treatment. Given the many limitations of direct detection of Bb, indirect detection represented by serum antibody testing occupies an important position in the diagnosis of LD. Serum antibody testing is simple to operate, and the process is easy to standardize. At present, all guidelines explicitly recommend against serologic testing in patients with typical EM. Instead, serological diagnosis should be performed exclusively in patients without typical EM who present with clinical suspicion of LD [5]. The enzyme immunoassay (EIA), indirect immunofluorescence assay (IFA), and western blot (WB) techniques are the most commonly utilized serum antibody detection methods. However, the combination of different detection methods with various antigens to detect different types of antibodies results in a relatively complex array of combinations that can create certain difficulties for clinical decision-making. In addition, in serological diagnostics in North America and Europe there are differences, linked to the fact that in America *Borrelia burgdorferi* sensu stricto (Bbss) is highly prevalent (B31 strain), while in Europe there are at least seven species of *Borreliae*, which can cause LD, and serology is more complex; one of the proposals concerns the use of chimeric antigens [6]. Finally, it should also be considered that in Asia *Ixodes persulcatus* transmits *Borrelia garinii*, *B. afzelii*, and *B. valaisiana*, but not Bbss, which is instead by far prevalent in North America. Therefore, we performed network meta-analysis (NMA) to systematically summarize and compare the diagnostic efficacy of various methods for LD and rank them, in the hope of providing some evidence-based medical support and references for clinical decision-making in LD.

## 2. Methods

This study was registered with PROSPERO, no. CRD42022378326 (https://www.crd.york.ac.uk/PROSPERO/ (accessed on 15 May 2025)). This NMA was carried out in accordance with the Preferred Reporting Items for Systematic Reviews and Meta-Analyses (PRISMA) statement [7].

### 2.1. Inclusion and Exclusion Criteria

The inclusion criteria were (1) studies on diagnostic accuracy that applied EIA, EIA combined with WB, or IFA for detecting serum antibodies to diagnose LD; (2) the study subjects included both adults and children, and the types of antibodies detected were IgM or IgG; (3) the gold standard for diagnosing LD was the comprehensive evaluation by physicians based on clinical manifestations combined with laboratory tests or the use of internationally recognized methods; (4) availability of complete data for analysis, and each study should have included true positive (TP), false positive (FP), true negative (TN), and false negative (FN) values; and (5) no restrictions on language of publication. The exclusion criteria were (1) studies with incomplete data (those missing any one of the TP, FP, FN, or TN values); (2) diagnostic tests with <3 references; and (3) the same diagnostic method used in combination with two or more antigens.

### 2.2. Search Strategies

Studies related to the serological diagnosis of LD were searched for in the Cochrane Library and PubMed databases from 1976 until June 2024. The search strategies are shown in Supplementary Table S1.

### 2.3. Screening and Data Extraction

After searching using the aforementioned strategy, an initial screening was conducted by reading the literature titles and abstracts, followed by a detailed full-text review for further screening. Literature that met the above conditions was included. We extracted information such as authors; publication year; country; type of study; number of cases; age and sex of study subjects; clinical manifestations; diagnostic methods; and values of TP, FP, FN, and TN. Two reviewers assessed all the included studies independently (Weijiang Ma and J. Li). Any disagreements were addressed via discussion with a third reviewer (F.K. Bao or A.H. Liu) until a consensus was reached.

### 2.4. Evaluation of Literature Quality

We conducted a methodological quality assessment of all included studies by using the Quality Assessment of Diagnostic Accuracy Studies-Comparative tool (QUADAS-C). QUADAS-C is a tool developed by Bada et al. [8] of Amsterdam University in the Netherlands in 2021, which is specially used to evaluate the risk of bias in studies comparing the accuracy of various diagnostic tests. The quality assessment was implemented using RevMan 5.3 (The Nordic Cochrane Center, Copenhagen, Denmark).

### 2.5. Statistical Analyses

#### 2.5.1. Traditional Meta-Analysis

For the serological diagnostic methods involved in the 52 included studies, we first conducted a traditional meta-analysis for each method to pool the diagnostic efficacy of each method. We used the Meta-disc 2.0 web version (https://ciberisciii.shinyapps.io/MetaDiSc2/ (accessed on 15 May 2025)) to perform a traditional meta-analysis on each diagnostic method [9]. The values of TP (a), FP (b), FN (c), and TN (d) were uploaded to the web page to calculate the pooled sensitivity, specificity, positive likelihood ratio (PLR), and negative likelihood ratio (NLR), respectively. Heterogeneity tests were conducted using *I*^2^ tests. When *I*^2^ > 50%, significant heterogeneity was considered to exist. Threshold effect analysis was conducted using Spearman’s correlation coefficient. For diagnostic methods with significant threshold effects, the aforementioned consolidation of individual indicators was abandoned.

#### 2.5.2. NMA

After pooling the diagnostic efficacy of each method, we conducted the NMA to compare the diagnostic efficacy of different methods. Considering the threshold effect of some diagnostic methods, we gave up the merger and comparison of single indicators (such as sensitivity and specificity), chose to fit the receiver operator characteristic (ROC) curve, and calculated the Q* index of the curve. The Q* index is defined as the point on the ROC curve where sensitivity equals specificity, and the Q-index is invariant to heterogeneity [10]. Q* index is the best statistical target to reflect the overall diagnostic value, which is the point closest to the ideal top-left corner of the summary receiver operating curve (SROC) space [11] and is in the range 0–1 (1 indicates better test performance) [12]. Then the Q* index values are graded accordingly and used to compare various diagnostic tests. First, we calculated the diagnostic odds ratio (DOR) for each diagnostic test with the following formula: DOR = (a × d)/(b × c). Subsequently, the calculated DOR values were used to compute the Q* index using the following formula: Q* index = √DOR/(1 + √DOR).

We use a frequentist approach to conduct the NMA. Since the TP (a), FP (b), FN (c), and TN (d) values for all diagnostic tests were calculated with reference to a common gold standard (the comprehensive evaluation), we assigned a Q* index value of 1.00 to this gold standard. Using this common gold standard as a medium and bridge, indirect comparisons between various diagnostic tests could be facilitated. Therefore, we used a consistency model for data analysis. Odds ratios (ORs) were used to report the effect size for assessing the Q* index, a funnel plot was used to analyze publication bias, and the surface under the cumulative ranking curve (SUCRA) indicated the likelihood of each diagnostic test being the best diagnostic method. The Q* index values of the diagnostic tests were ranked in ascending order according to their SUCRA values. All the above operations were implemented using the network package in Stata 15.0 (StataCorp LP, College Station, TX, USA).

## 3. Results

### 3.1. Process and Results of Literature Screening

**Figure 1:**
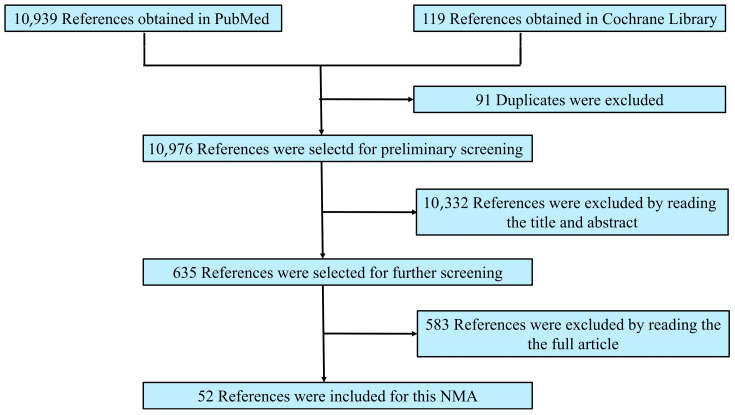
Reference screening flow diagram.

Following the aforementioned search strategy, we retrieved 10,939 articles from the PubMed database and 119 articles from the Cochrane Library. After excluding duplicates and ineligible studies, we finally included 52 articles (Figure 1).

### 3.2. Characteristics and Risk of Bias of Included Literature

**Figure 2:**
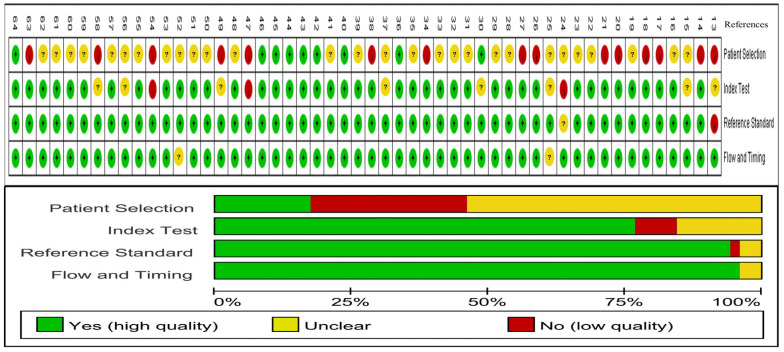
Graph of bias risk assessment for included studies [13,14,15,16,17,18,19,20,21,22,23,24,25,26,27,28,29,30,31,32,33,34,35,36,37,38,39,40,41,42,43,44,45,46,47,48,49,50,51,52,53,54,55,56,57,58,59,60,61,62,63,64].

The majority of the 52 included studies were retrospective, involving a total study population of 18,103 patients, of whom 5154 had LD. Apart from the gold standard, a total of 14 diagnostic tests were involved: whole-cell antigen (WCA) EIA for IgM, WCA EIA for IgG, flagella EIA for IgM, flagella EIA for IgG, VlsE EIA for IgM, VlsE EIA for IgG, C6 EIA, IFA for IgM, IFA for IgG, WCA WB for IgM, WCA WB for IgG, standard two-tiered test (STTT), modified two-tiered test (MTTT), and DbpB EIA for IgG. The age range of the patients in the reported data was 0–93 years. Other characteristics are detailed in Supplementary Table S2 [13,14,15,16,17,18,19,20,21,22,23,24,25,26,27,28,29,30,31,32,33,34,35,36,37,38,39,40,41,42,43,44,45,46,47,48,49,50,51,52,53,54,55,56,57,58,59,60,61,62,63,64]. Some studies had a high risk of bias in patient selection, while the overall risk of bias in the remaining studies was generally acceptable (Figure 2).

### 3.3. Outcomes

#### 3.3.1. Traditional Meta-Analysis Results

The threshold effect test results showed significant threshold effects for flagella EIA for IgM, C6 EIA, IFA for IgM, WCA WB for IgM, and DbpB EIA for IgG. Therefore, we discarded the single indicator pooling for these tests. Meta-analysis results for other diagnostic tests are detailed in Table 1. The SROC for each diagnostic test is shown in Supplementary Figures S1–S14.

#### 3.3.2. NMA

**Figure 3:**
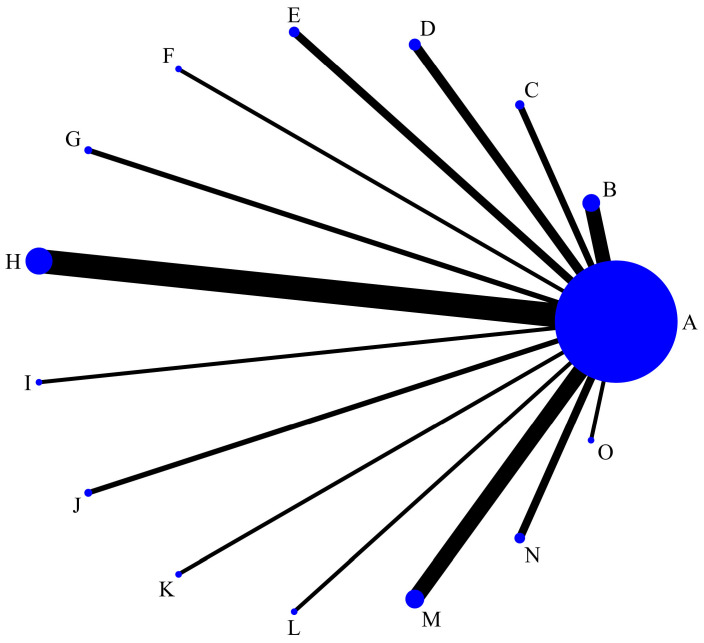
Network meta-analysis graphs of the Q* index of each different diagnostic test. A = gold Standard; B = whole-cell antigen (WCA) enzyme immunoassay (EIA) for IgM; C = WCA EIA for IgG; D = flagella EIA for IgM; E = flagella EIA for IgG; F = VlsE EIA for IgM; G = VlsE EIA for IgG; H = C6 EIA; I = indirect immunofluorescence assay (IFA) for IgM; J = IFA for IgG; K = WCA Western blot (WB) for IgM; L = WCA WB for IgG; M = standard two-tiered testing; N = modified two-tiered testing; O = DbpB EIA for IgG.

Figure 3 presents the NMA comparing the Q* index values of various diagnostic tests. All diagnostic tests were directly compared to the gold standard, with C6 EIA having the most comparisons against the gold standard.

**Figure 4:**
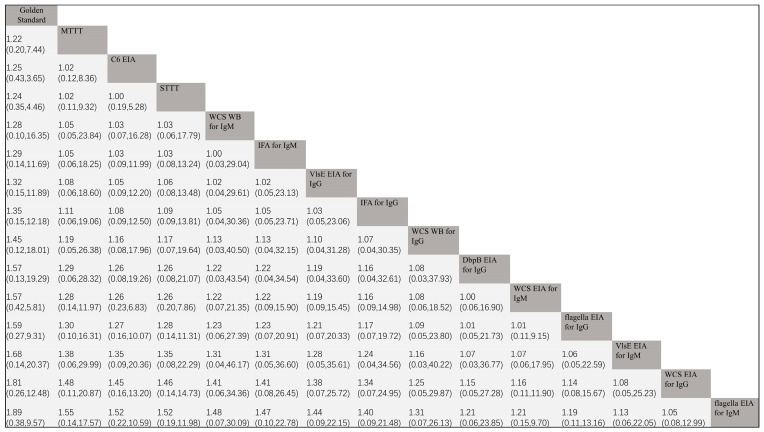
The network meta-analysis results of comparing the Q* index of various diagnostic tests. WCA = whole-cell antigen; EIA = enzyme immunoassay; IFA = indirect immunofluorescence assay; WB = Western blot; STTT = standard two-tiered testing; MTTT = modified two-tiered testing.

All comparisons between diagnostic tests were indirect, with the gold standard serving as a “mediator” and “bridge.” The results of the pairwise comparisons of Q* index values are shown in Figure 4. Because we artificially assigned the Q* index value of the gold standard to 1 in advance, the Q* index value of the gold standard naturally exceeds that of all diagnostic tests. The NMA results showed that, excluding the gold standard, the Q* index of MTTT was greater than all other diagnostic tests, indicating a higher overall diagnostic value. Upon comparing C6 EIA with STTT, both were nearly equivalent (OR: 1.00, 95% CI: 0.19–5.28). The Q* index of flagella EIA for IgM was the smallest among all diagnostic tests, indicating the least diagnostic value.

**Figure 5:**
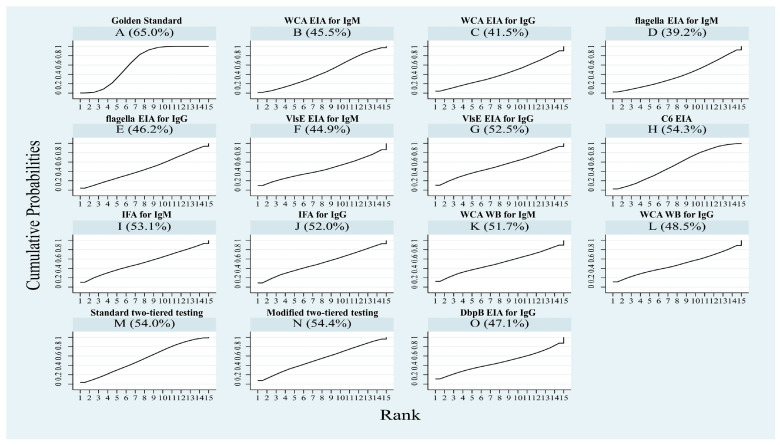
The SUCRA values for the ranking of Q* index of various diagnostic tests. WCA = whole-cell antigen; EIA = enzyme immunoassay; IFA = indirect immunofluorescence assay; WB = Western blot.

The SUCRA value is the area under the cumulative ranking curve, ranging from 0 to 1. The closer the value is to 1, the higher the probability that the Q* index of the diagnostic test is in a higher position in all rankings. Based on the SUCRA values, the Q* index of all diagnostic tests was ranked from highest to lowest, excluding the gold standard (Figure 5): MTTT (54.4%) > C6 EIA (54.3%) > STTT (54.0%) > WCA WB for IgM (51.7%) > IFA for IgM (53.1%) > VlsE EIA for IgG (52.5%) > IFA for IgG (52.0%) > WCA WB for IgG (48.5%) > DbpB EIA for IgG (47.1%) > WCA EIA for IgM (45.5%) > flagella EIA for IgG (46.2%) > VlsE EIA for IgM (44.9%) > WCA EIA for IgG (41.5%) > flagella EIA for IgM (39.2%). MTTT has the highest probability of optimal diagnostic value.

## 4. Discussion

Although culture is the gold standard for diagnosis of most infectious diseases, it is limited by a long culture cycle and low positive rate of Bb [65]. The positivity rate of PCR for spirochete detection can exhibit high variability depending on the tissue sample (e.g., cerebrospinal fluid, blood, synovial fluid) and the method used (e.g., conventional PCR, nested PCR, quantitative PCR) [66]. Furthermore, because of the multitude of target genes selected for detection and the varied genospecies of Bb causing LD in humans, PCR diagnosis for LD often lacks standardization. This has resulted in limitations to its clinical application [65,67].

Antibody detection, as a representative indirect test, is important in the diagnosis of LD. Typical EM can be clinically diagnosed directly without laboratory tests, but serological testing is required in the case of suspected LD [68,69]. Because of the different antigens used, the different test methods used to detect antibodies, and the different types of antibodies detected (IgM or IgG), there is some confusion among clinicians in the selection of serological tests for suspected LD cases. Currently, 24 species of the *Borrelia* Lyme group have been isolated, of which nine are pathogenic for humans [70]. The last one is *Borrelia Maritima*, non-pathogenic for humans, isolated by Margos on the California coast [71]. Therefore, we hope that through this NMA, we can rank the diagnostic value of various serological tests to aid with clinical decision-making. In assessing the diagnostic value, we hoped to identify an overall diagnostic test that excels in both sensitivity and specificity. Consequently, we opted for the Q* index as our evaluation metric, which reflects the comprehensive diagnostic performance, rather than relying solely on any individual evaluation index [11]. The Q* index offers a comprehensive assessment, incorporating both sensitivity and specificity, thereby enabling a more accurate judgment of the overall diagnostic worthiness of the test [72]. Additionally, it is noteworthy that we employed the gold standard as a “medium” and “bridge” to enable indirect comparisons among various diagnostic tests. As such, we artificially assigned a Q* index value of 1 to the gold standard. In this study, the gold standard serves solely as a reference and benchmark; it does not hold practical significance in direct comparison with other diagnostic tests.

Currently, most guidelines and recommendations from the CDC endorse the use of an STTT strategy, which involves an initial screening with EIA, followed by a confirmatory WB test for positive or equivocal EIA results. Only when both EIA and WB are positive will the entire STTT test be judged as positive, so STTT has the advantage of high specificity [5]. The positive criterion for IgM WB is defined as the presence of at least two positive bands out of three, including p21 (OspC), p39 (BmpA), and p41 (flagellin B). By contrast, for IgG WB, the positive criterion is set as the presence of at least five positive bands out of 10, encompassing p17–18 (DbpA), p21–25 OspC (usually p23 in the USA and p25 in Europe), p28, p30, p39 (BmpA), p41 (flagellin), p45, p58, p66, and p93 [73]. The widespread adoption of STTT has significantly contributed to the diagnosis of LD. In our study, the Q* index value of STTT ranks third, a finding that reinforces the diagnostic value. However, the limitations of STTT have become increasingly apparent. First, the WB procedure is cumbersome and time-consuming, with a high degree of subjectivity in result interpretation, making it difficult to standardize. Second, STTT has low sensitivity in early-stage LD cases, with an average sensitivity of only about 50% for EM, which is not conducive to early disease diagnosis [67]. These are the limitations that prevent STTT from being the best diagnostic strategy.

The WCA composition is complex, the quality is unstable, and cross-reactions often occur, so its diagnostic value is not ideal. Our results showed that, except for WCA WB for IgM, the Q* index values of the other three tests based on WCA all ranked in the middle and lower positions.

Recombinant or synthetic antigens have high purity, clear components, and stable quality, which make them conducive to standardization, e.g., a peptide (C6 peptide) containing a highly conserved sequence of 25 amino acids extracted from the invariable region 6 of VlsE protein [74]. Numerous studies have shown that the C6 peptide as an antigen has higher sensitivity than STTT, but at the expense of some specificity [73]. Our NMA results also show that the SUCRA value of the Q* index for C6 EIA ranks second, and there was essentially no significant difference when compared with STTT (OR: 1.00, 95% CI: 0.19–5.28), which has a good comprehensive diagnostic value. However, considering that the specificity of C6 EIA is slightly inferior to STTT, some scholars have proposed an MTTT strategy, which uses WCA EIA as the first-tier assay and C6 EIA as the second-tier assay. Multiple studies have compared the diagnostic value of STTT and MTTT, and the results consistently show that MTTT has higher sensitivity for early-stage cases without compromising specificity than STTT, demonstrating satisfactory diagnostic value [4,74]. The CDC also recommends MTTT as an alternative strategy to STTT for the diagnosis of LD [75]. Our NMA results also showed that MTTT ranks first in the Q* index, having the best diagnostic value among all the diagnostic tests, which is consistent with most current studies. Therefore, MTTT can be expected to replace STTT.

In this study, flagella EIA for IgM came in last place, and flagella EIA for IgG was the fourth to last item, which suggests that the diagnostic value of flagella as an antigen is poor. Flagella, as the main antigen of spirochetes, can produce strong IgM and IgG reactions a few days after human infection. However, flagella have strong cross-reactions with other bacterial antigens and antigens in mammalian tissues, which make them a relatively sensitive but poorly specific diagnostic antigen, resulting in lower overall diagnostic value [5,73]. The ranking difference between VlsE EIA for IgG and VlsE EIA for IgM is significant, indicating that VlsE as an antigen may be acceptable for the diagnosis of late-stage LD. This result is consistent with the research conclusions of Chmielewska–Badora et al. [76].

IFA, as an earlier method applied to LD diagnosis, uses complete spirochetes as antigens, which has a higher possibility of cross-reactions. Coupled with the strong subjectivity in result interpretation, we anticipate that the diagnostic value of IFA may not be ideal. Surprisingly, the results of this study showed that the Q* index ranking of IFA comes up in the upper middle position among all tests, which was better than expected.

## 5. Limitations

Our study also has some limitations. First, the number of studies involved in some diagnostic tests was still small, which may have led to deviations between the NMA results and actual results. Hence, caution is needed when interpreting and applying the results. In addition, limited by the incompleteness of the available reported data in some included studies, we were unable to stage the included LD patients, which makes the results of NMA only a general conclusion for LD. Therefore, it was not possible to accurately compare the diagnostic value of different diagnostic tests in different stages of LD. Thirdly, the differences in the Lyme Borreliae species in North America, Europe, and Asia should also be considered. Finally, some of the included studies showed a high risk of bias in patient selection, which may have exaggerated or undervalued the results of some diagnostic tests.

## 6. Conclusions

This study could provide a reference for the decision-making of the LD serum diagnosis scheme. This study comprehensively confirmed the diagnostic value of the current mainstream method, STTT, for LD and found that the diagnostic value of C6 EIA and MTTT was no less than STTT. MTTT had the highest diagnostic value in all tests, and its operation was more convenient and easy to standardize and has the most potential to replace STTT. The diagnostic accuracy of flagella EIA for IgM was the worst, and flagella EIA for IgG was also unsatisfactory. Thus, it is not a wise choice to use flagella as an antigen.

## Figures and Tables

**Table 1:** Results of conventional meta-analysis.

Test	Number of Studies	Threshold Effect		Heterogeneity		Point Estimate (95% *CI*)			
		R Value	*p* Value	*I* ^2^	*p* Value	Pooled Sen	Pooled Spe	Pooled PLR	Pooled NLR
WCA EIA for IgM	11	−0.52	0.61	98%	0.00	0.57 (0.43, 0.69)	0.95 (0.86, 0.98)	11.56 (4.33, 30.86)	0.46 (0.40, 0.61)
WCA EIA for IgG	5	0.60	0.28	99%	0.00	0.73 (0.65, 0.79)	0.80 (0.44, 0.96)	3.67 (0.97, 14.00)	0.34 (0.22, 0.52)
flagella EIA for IgM	7	0.821	0.02	86%	0.00	/	/	/	/
flagella EIA for IgG	6	−0.79	0.62	86%	0.00	0.65 (0.56, 0.72)	0.92 (0.85, 0.96)	7.98 (4.25, 15.00)	0.38 (0.31, 0.47)
VlsE EIA for IgM	3	0.500	0.67	92.0%	0.00	0.46 (0.36, 0.56)	0.94 (0.79, 0.99)	8.02 (1.94, 33.13)	0.57 (0.46, 0.70)
VlsE EIA for IgG	4	−0.28	0.40	98%	0.00	0.85 (0.52, 0.97)	0.95 (0.83, 0.99)	16.89 (4.92, 58.03)	0.16 (0.04, 0.64)
C6 EIA	17	−0.58	0.01	100.0%	0.00	/	/	/	/
IFA for IgM	4	−1.000	0.00	33%	0.11	/	/	/	/
IFA for IgG	4	−0.200	0.800	89%	0.00	0.75 (0.68, 0.80)	0.94 (0.87, 0.95)	12.56 (2.47, 63.82)	0.27 (0.21, 0.35)
WCA WB for IgM	3	1.000	0.000	0.0%	0.71	/	/	/	/
WCA WB for IgG	3	−0.500	0.667	0.0%	0.42	0.44 (0.30, 0.60)	0.98 (0.89, 1.00)	24.71 (3.51, 174.01)	0.57 (0.43, 0.75)
STTT	12	0.000	1.000	92%	0.000	0.66 (0.54, 0.76)	0.99 (0.98, 0.99)	63.16 (36.27, 110.01)	0.35 (0.25, 0.48)
MTTT	6	−0.200	0.704	96%	0.000	0.73 (0.61, 0.82)	0.98 (0.96, 0.99)	40.81 (19.58, 85.06)	0.28 (0.19, 0.41)
DbpB EIA for IgG	3	−1.000	0.000	80.7%	0.0057	/	/	/	/

Notes: WCA = whole-cell antigen; EIA = enzyme immunoassay; IFA = indirect immunofluorescence assay; WB = Western blot; STTT = standard two-tiered testing; MTTT = modified two-tiered testing; Sen = sensitivity; Spe = specificity; PLR = positive likelihood ratio; NLR = negative likelihood ratio; *CI* = confidence interval.

## Data Availability

The original contributions presented in this study are included in the article/Supplementary Material. Further inquiries can be directed to the corresponding author(s).

## Supplementary Materials

The following supporting information can be downloaded at: https://www.mdpi.com/article/10.3390/pathogens14080784/s1; Table S1: The search strategy; Table S2: Characteristics of included studies; Figure S1: Summary receiver operating curve (SROC) of the WCA EIA for IgM. WCA = Whole cell antige; Figure S2: Summary receiver operating curve (SROC) of the WCA EIA for IgG. WCA = Whole cell antige; Figure S3: Summary receiver operating curve (SROC) of the flagella EIA for IgM; Figure S4: Summary receiver operating curve (SROC) of the flagella EIA for IgG; Figure S5: Summary receiver operating curve (SROC) of the VlsE EIA for IgM; Figure S6: Summary receiver operating curve (SROC) of the VlsE EIA for IgG; Figure S7: Summary receiver operating curve (SROC) of the C6 EIA; Figure S8: Summary receiver operating curve (SROC) of the IFA for IgM; Figure S9: Summary receiver operating curve (SROC) of the IFA for IgG; Figure S10: Summary receiver operating curve (SROC) of the WCA WB for IgM. WCA = Whole cell antige; WB = Western blot; Figure S11: Summary receiver operating curve (SROC) of the WCA WB for IgG. WCA = Whole cell antige; WB = Western blot; Figure S12: Summary receiver operating curve (SROC) of the STTT; Figure S13: Summary receiver operating curve (SROC) of the MTTT; Figure S14: Summary receiver operating curve (SROC) of the DbpB EIA for IgG.
