# Supplementary material for: Comparison of the Serodiagnostic Accuracy Tests for Lyme Disease in Adults and Children: A Network Meta-Analysis
Source: Pathogens. 2025 Aug 6;14(8):784. doi: 10.3390/pathogens14080784 (PMC12389093; doi:10.3390/pathogens14080784)
Supplement: Supplementary file 1 [file pathogens-14-00784-s001.zip › Supplementary Figure legends.pdf]

Supplementary Figure legends:

Supplementary Figure S1 Summary receiver operating curve (SROC) of the WCA EIA for IgM. WCA=Whole cell antige

Supplementary Figure S2 Summary receiver operating curve (SROC) of the WCA EIA for IgG. WCA=Whole cell antige

Supplementary Figure S3 Summary receiver operating curve (SROC) of the flagella EIA for IgM

Supplementary Figure S4 Summary receiver operating curve (SROC) of the flagella EIA for IgG

Supplementary Figure S5 Summary receiver operating curve (SROC) of the VlsE EIA for IgM

Supplementary Figure S6 Summary receiver operating curve (SROC) of the VlsE EIA for IgG

Supplementary Figure S7 Summary receiver operating curve (SROC) of the C6 EIA

Supplementary Figure S8 Summary receiver operating curve (SROC) of the IFA for IgM. IFA= Indirect immunofluorescence assay

Supplementary Figure S9 Summary receiver operating curve (SROC) of the IFA for IgG. IFA= Indirect immunofluorescence assay

Supplementary Figure S10 Summary receiver operating curve (SROC) of the WCA WB for IgM. WCA=Whole cell antige; WB= Western blot

Supplementary Figure S11 Summary receiver operating curve (SROC) of the WCA WB for IgG. WCA=Whole cell antige; WB= Western blot

Supplementary Figure S12 Summary receiver operating curve (SROC) of the STTT. STTT= Standard two-tiered testing

Supplementary Figure S13 Summary receiver operating curve (SROC) of the MTTT. MTTT= Modified two-tiered testing

Supplementary Figure S14 Summary receiver operating curve (SROC) of the DbpB EIA for IgG
