# Supplementary material for: Comparison of the Serodiagnostic Accuracy Tests for Lyme Disease in Adults and Children: A Network Meta-Analysis
Source: Pathogens. 2025 Aug 6;14(8):784. doi: 10.3390/pathogens14080784 (PMC12389093; doi:10.3390/pathogens14080784)
Supplement: Supplementary file 1 [file pathogens-14-00784-s001.zip › Supplementary Tables.pdf]

Supplementary Table S1 The search strategy

Step	Search strategy
#1	(Lyme disease) OR (Lyme borreliosis) OR (borrelia burgdorferi) OR (Lyme neuroborreliosis) OR (Lyme arthritis) OR (Lyme Carditis)
#2	(diagnose) OR (diagnosis) OR (serological diagnosis) OR (serum diagnosis) OR (serodiagnose) OR (serology)
#3	(OspA) OR (OspB) OR (OspC) OR (BmpA) OR (flagellin) OR (flagellum) OR (FlaA) OR (FlaB) OR (pepC10) OR (VlsE) OR (C6) OR (IR6) OR (DbpA) OR (DbpB) OR (BBK07) OR (BBK32) OR (P83) OR (P58) OR (P39) OR (P30) OR (P23) OR (P17) OR (P66) OR (P32) OR (P36) OR (P39) OR (P41)
#4	(enzyme linked immunosorbent assay) OR (ELISA) OR (enzyme immunoassay) OR (EIA) OR (western blot) OR (immunoblotting) OR (enzyme linked fluorescent assay) OR (ELFA)
#5	#1 AND (#2 OR #3 OR #4)

Supplementary Table S2 Characteristics of included studies

Study ID	Author (publication year)	Country	Sample size (M/F)	Age (years)	Test	TP	FP	FN	TN
1	Kaiser, 1999[13]	Germany	216(NR)	NR	WCA EIA for IgM	51	8	45	112
					WCA EIA for IgG	75	93	21	27
2	Wilske, 1993[14]	Germany	276(NR)	NR	WCA EIA for IgM	61	4	73	138
					WCA EIA for IgG	81	4	53	138
					flagella EIA for IgM	48	6	86	136
					flagella EIA for IgG	95	8	39	134
					IFA for IgM	30	4	104	138
					IFA for IgG	102	4	32	138
3	Qiu, 2000[15]	USA	56(NR)	NR	WCA EIA for IgM	23	6	10	17
4	Magnarelli, 1988[16]	USA	179(NR)	NR	WCA EIA for IgM	86	32	16	45
					WCA EIA for IgG	73	24	22	44
5	Rauer, 1998[17]	Germany	146(NR)	NR	WCA EIA for IgM	47	10	57	32
6	Kaiser, 1999[18]	Germany	163(NR)	NR	WCA EIA for IgM	40	8	43	72
					WCA EIA for IgG	67	26	16	54
7	Fikrig, 1992[19]	USA	49(NR)	NR	WCA EIA for IgM	6	0	19	12
8	Gerber, 1995[20]	USA	132(NR)	1-18	WCA EIA for IgM	23	0	59	50
9	Lencáková, 2008[21]	Slovakia	134(NR)	NR	WCA EIA for IgM	34	1	4	37
					WCA EIA for IgG	39	1	21	35
					IFA for IgM	21	1	11	53
					IFA for IgG	35	1	19	39
10	Berardi, 1988[22]	USA	163(NR)	NR	WCA EIA for IgM	20	4	10	129
11	Padula, 1994[23]	USA	150(NR)	NR	WCA EIA for IgM	43	0	31	76
					WCA western blot for IgM	41	3	33	73
					WCA western blot for IgG	41	3	33	73
12	Mathiesen, 1998[24]	USA	348(NR)	6-83	flagella EIA for IgM	98	15	112	123
13	Smismans, 2006[25]	Netherlands	85(NR)	NR	flagella EIA for IgM	23	9	13	31
					flagella EIA for IgG	18	0	16	40
					C6 EIA	32	3	4	37
14	Panelius, 2001[26]	Finland	130(NR)	NR	flagella EIA for IgM	25	8	28	69
					flagella EIA for IgG	34	7	19	70

15	Karlsson, 1990[27]	Sweden	122(NR)	NR	flagella EIA for IgM	27	3	41	41
					flagella EIA for IgG	39	3	29	41
16	Magnarelli, 2000[28]	USA	101(NR)	NR	flagella EIA for IgM	9	19	8	65
					flagella EIA for IgG	6	11	9	72
17	Hansen, 1991[29]	Denmark	280(NR)	5-80	flagella EIA for IgM	69	10	81	72
					flagella EIA for IgG	112	19	38	63
18	Barstad, 2017[30]	Norway	210(93/117)	0.25-18	VlsE EIA for IgM	40	25	37	94
					VlsE EIA for IgG	66	35	11	84
19	Marangoni, 2008[31]	Italy	466(NR)	8-82	VlsE EIA for IgM	36	29	30	371
					VlsE EIA for IgG	37	14	29	386
20	Bacon, 2003[32]	USA	839(NR)	NR	VlsE EIA for IgM	102	6	178	553
					VlsE EIA for IgG	184	6	96	553
					C6 EIA	186	6	94	553
					Standard two-tiered testing	189	5	91	554
21	Peltomaa, 2004[33]	USA	133(NR)	4-74	VlsE EIA for IgG	47	4	0	82
					Standard two-tiered testing	47	2	0	86
22	Liang, 1999[34]	USA	386(NR)	NR	C6 EIA	184	2	26	174
23	Pomelova, 2015[35]	Russia	374(NR)	59.7 ± 14.8	C6 EIA	48	2	26	174
24	van Gorkom, 2021[36]	Netherlands	156(78/78)	49-51	C6 EIA	7	37	0	112
25	Pegalajar, 2018[37]	USA	42(26/16)	18-86	C6 EIA	13	4	1	24
26	Hahm, 2020[38]	USA	280(NR)	NR	C6 EIA	77	5	13	185
27	Marangoni, 2005[39]	Italy	319(NR)	NR	C6 EIA	25	9	20	265
28	Nigrovic, 2020[40]	USA	911(572/339)	4-11	C6 EIA	211	39	0	661
29	Rouhiainen, 2021[41]	Finland	1368(726/642)	0-93	C6 EIA	391	104	41	832
30	Riesbeck, 2007[42]	Sweden	157(72/85)	5-88	C6 EIA	27	7	1	122
31	Ledue, 2008[43]	USA	904(NR)	NR	C6 EIA	75	17	22	790
32	Hoeve-Bakker, 2022[44]	Netherlands	196(174/22)	5-83	C6 EIA	73	20	1	102
33	Tjernberg, 2007[45]	Sweden	484(NR)	NR	C6 EIA	110	31	112	222
34	Skarpaas, 2007[46]	Norway	128(NR)	NR	C6 EIA	66	23	2	37
35	Branda, 2017[47]	USA	1332(NR)	NR	C6 EIA	36	20	19	1257

					Modified two-tiered testing	21	7	34	1270
36	Wormser, 2013[48]	USA	2777(NR)	NR	C6 EIA	427	24	142	2184
					Standard two-tiered testing	293	12	276	2196
37	Mitchell, 1994[49]	USA	161(NR)	2-76	IFA for IgM	40	0	11	110
38	Craft, 1984[50]	USA	52(NR)	NR	IFA for IgM	9	0	3	40
					IFA for IgG	11	0	1	40
39	Artsob, 1990[51]	Canada	43(NR)	NR	IFA for IgG	7	17	1	18
40	Jain, 1996[52]	USA	25(NR)	3.5-77	WCA western blot for IgM	12	0	3	10
					WCA western blot for IgG	3	0	12	10
41	Ledue, 1996[53]	USA	112(38/74)	NR	WCA western blot for IgM	23	0	31	58
					WCA western blot for IgG	24	0	30	58
					Standard two-tiered testing	27	0	27	58
42	Snyder, 2017[54]	USA	66(NR)	NR	Standard two-tiered testing	1	0	1	2
43	Steere, 2008[55]	USA	331(NR)	NR	Standard two-tiered testing	67	2	53	209
44	Baarsma, 2020[56]	Netherlands	509(NR)	NR	Standard two-tiered testing	106	12	122	269
					Modified two-tiered testing	162	15	66	266
45	Eshoo, 2012[57]	USA	65(NR)	NR	Standard two-tiered testing	14	1	7	43
46	Molins, 2016[58]	USA	471(NR)	NR	Standard two-tiered testing	83	6	41	341
					Modified two-tiered testing	94	6	30	341
47	Lipsett, 2016[59]	USA	944(NR)	NR	Standard two-tiered testing	93	10	21	820
					Modified two-tiered testing	91	29	23	801
48	Branda, 2013[60]	USA	164(NR)	NR	Standard two-tiered testing	33	0	31	100
					Modified two-tiered testing	54	0	10	100
49	Pegalajar, 2018[61]	USA	471(NR)	NR	Standard two-tiered testing	82	1	42	346
					Modified two-tiered testing	97	8	27	339
50	Arnaboldi, 2014[62]	USA	312(NR)	NR	DbpB EIA for IgG	73	8	51	132
51	Heikkilä, 2002[63]	Germany	92(NR)	NR	DbpB EIA for IgG	40	1	12	39
52	Sillanpää, 2014[64]	Finland	93(NR)	2-17	DbpB EIA for IgG	17	7	23	46

Notes: WCA=Whole cell antigen; EIA=enzyme immunoassay; IFA=Indirect immunofluorescence assay; P= Prospective; R= Retrospective; NR= Not reported.
