# Supplementary figures and images for: Comparison of the Serodiagnostic Accuracy Tests for Lyme Disease in Adults and Children: A Network Meta-Analysis
Source: Pathogens. 2025 Aug 6;14(8):784. doi: 10.3390/pathogens14080784 (PMC12389093; doi:10.3390/pathogens14080784)

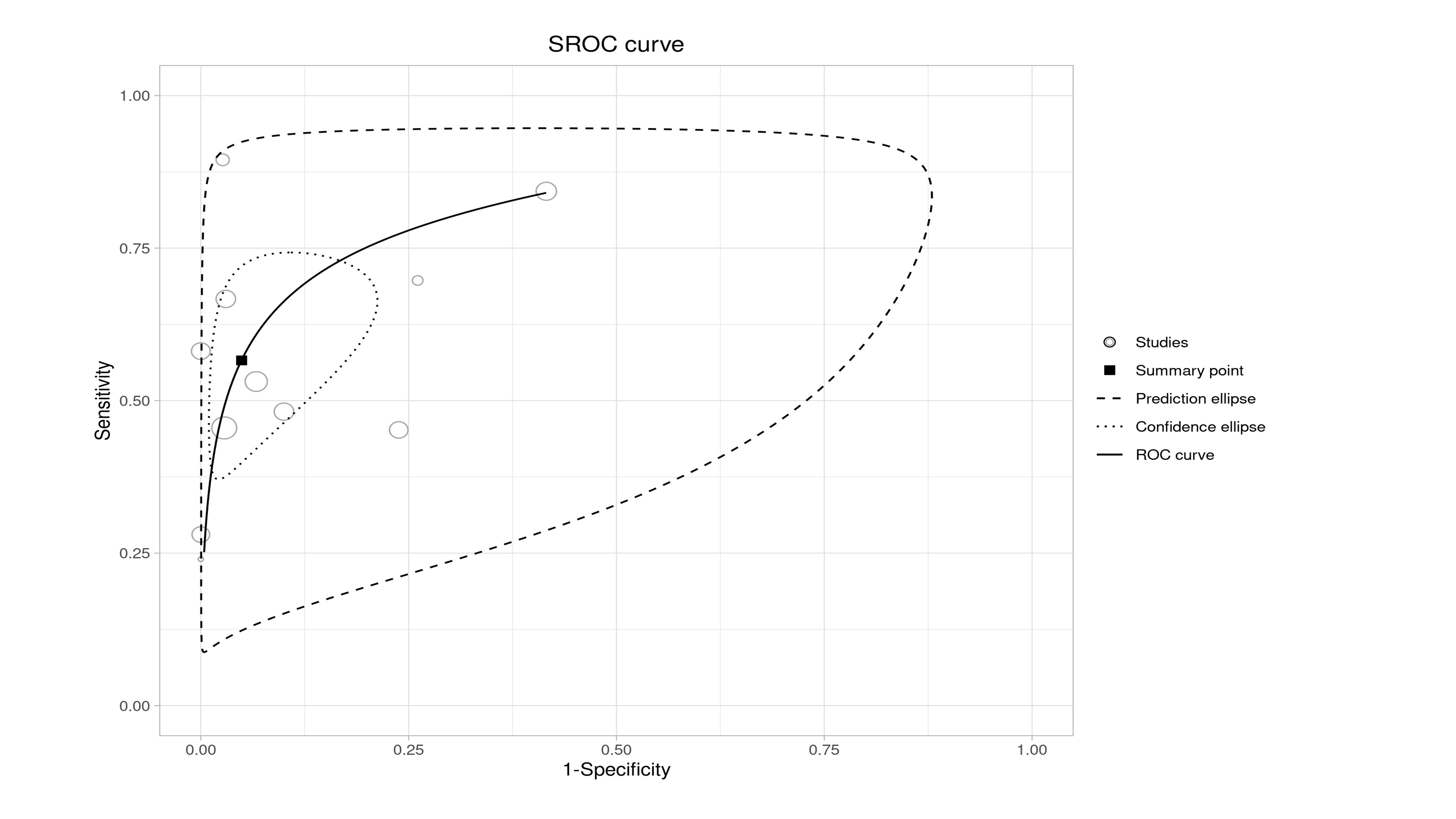

Supplement: Supplementary file 1 [file pathogens-14-00784-s001.zip › Supplementary Figure 1.jpg]

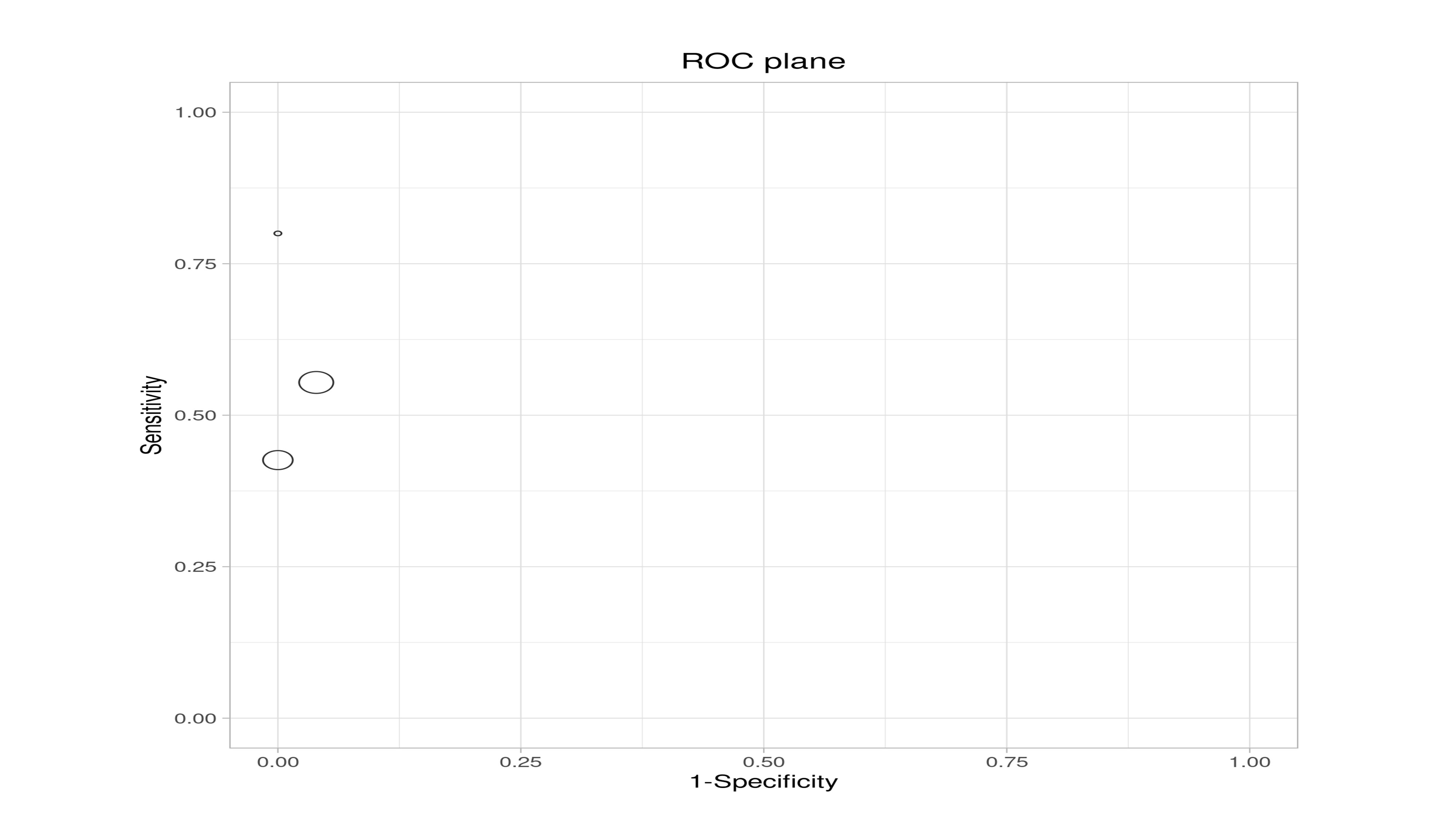

Supplement: Supplementary file 1 [file pathogens-14-00784-s001.zip › Supplementary Figure 10.jpg]

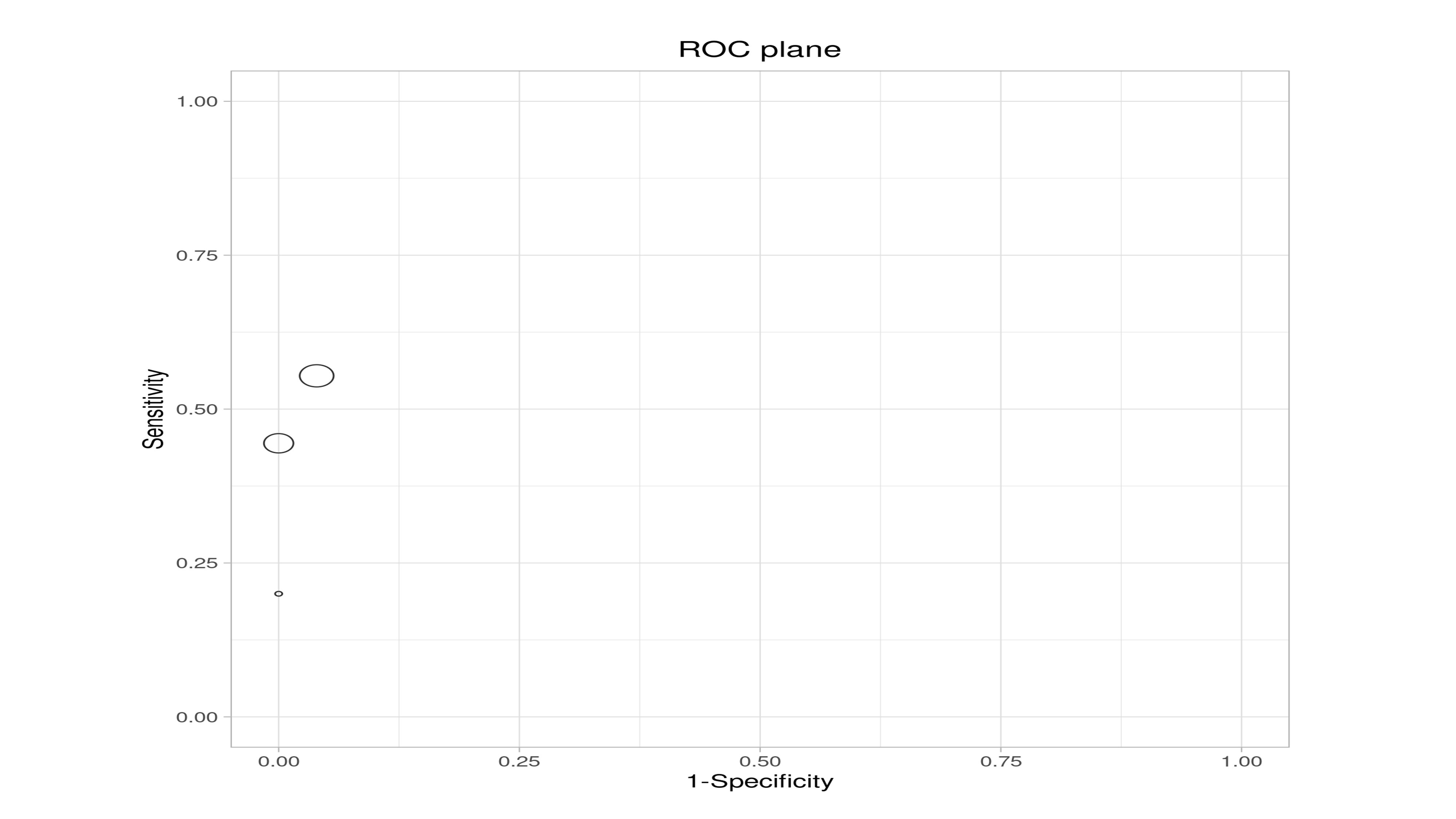

Supplement: Supplementary file 1 [file pathogens-14-00784-s001.zip › Supplementary Figure 11.jpg]

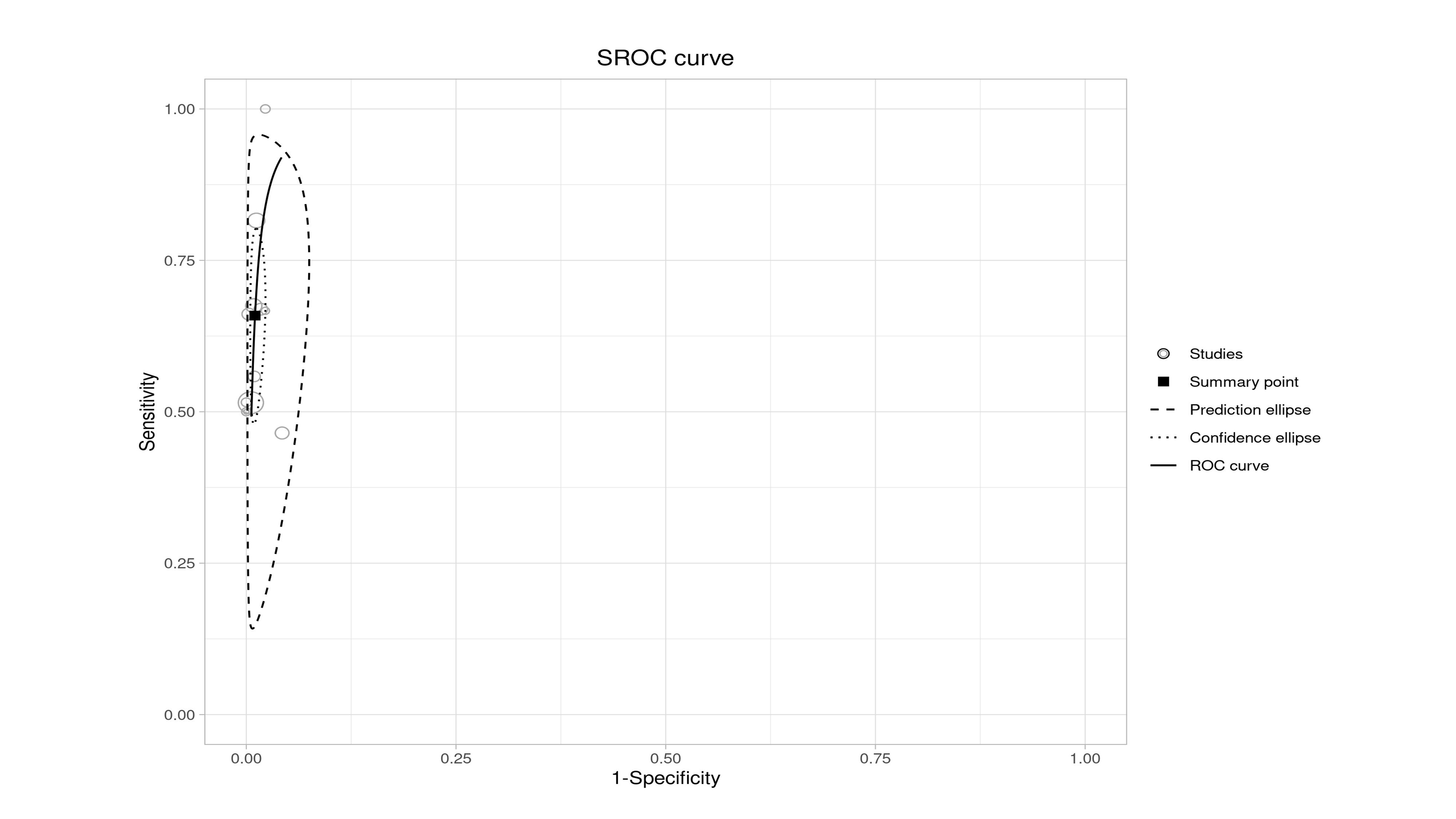

Supplement: Supplementary file 1 [file pathogens-14-00784-s001.zip › Supplementary Figure 12.jpg]

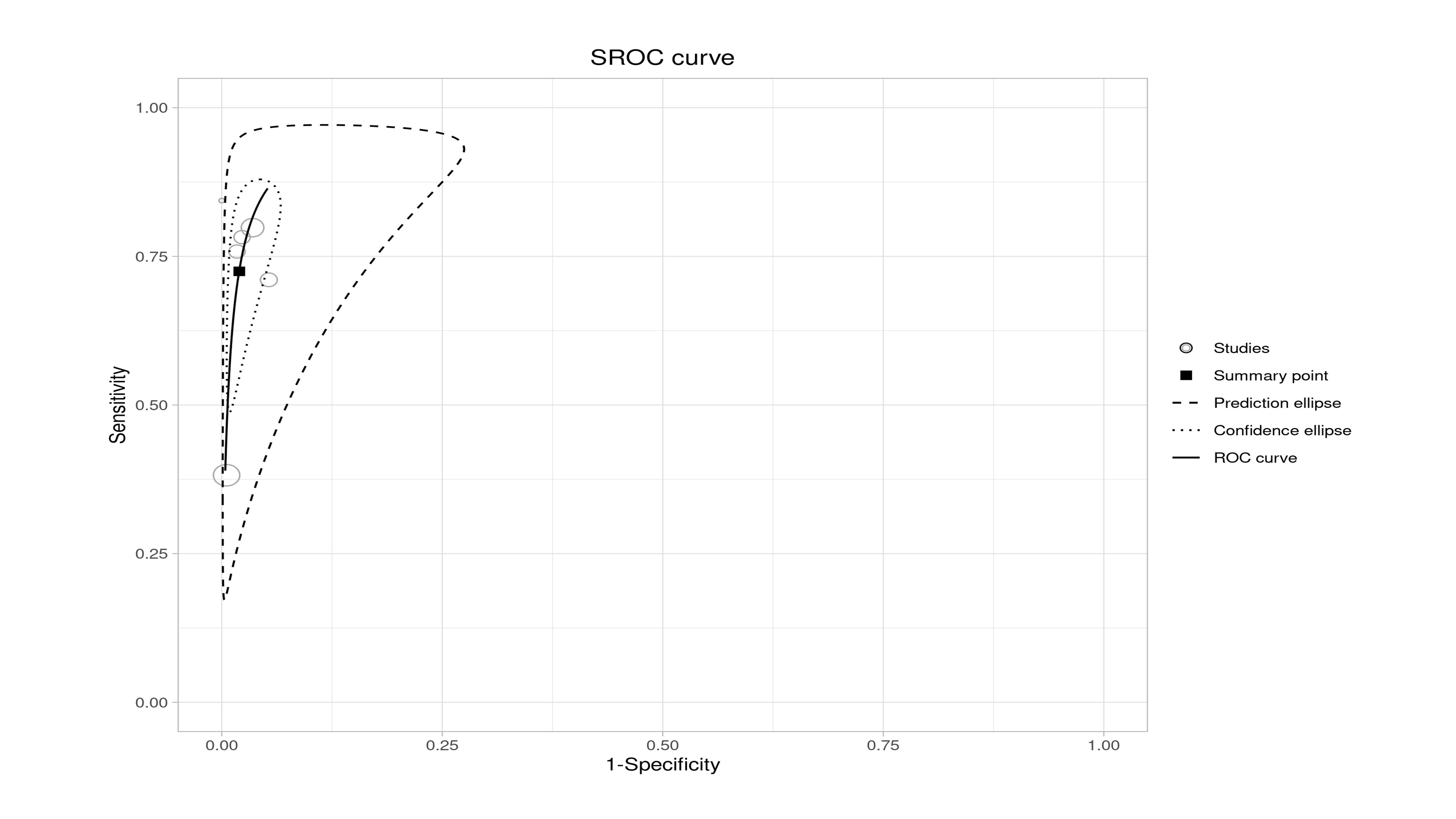

Supplement: Supplementary file 1 [file pathogens-14-00784-s001.zip › Supplementary Figure 13.jpg]

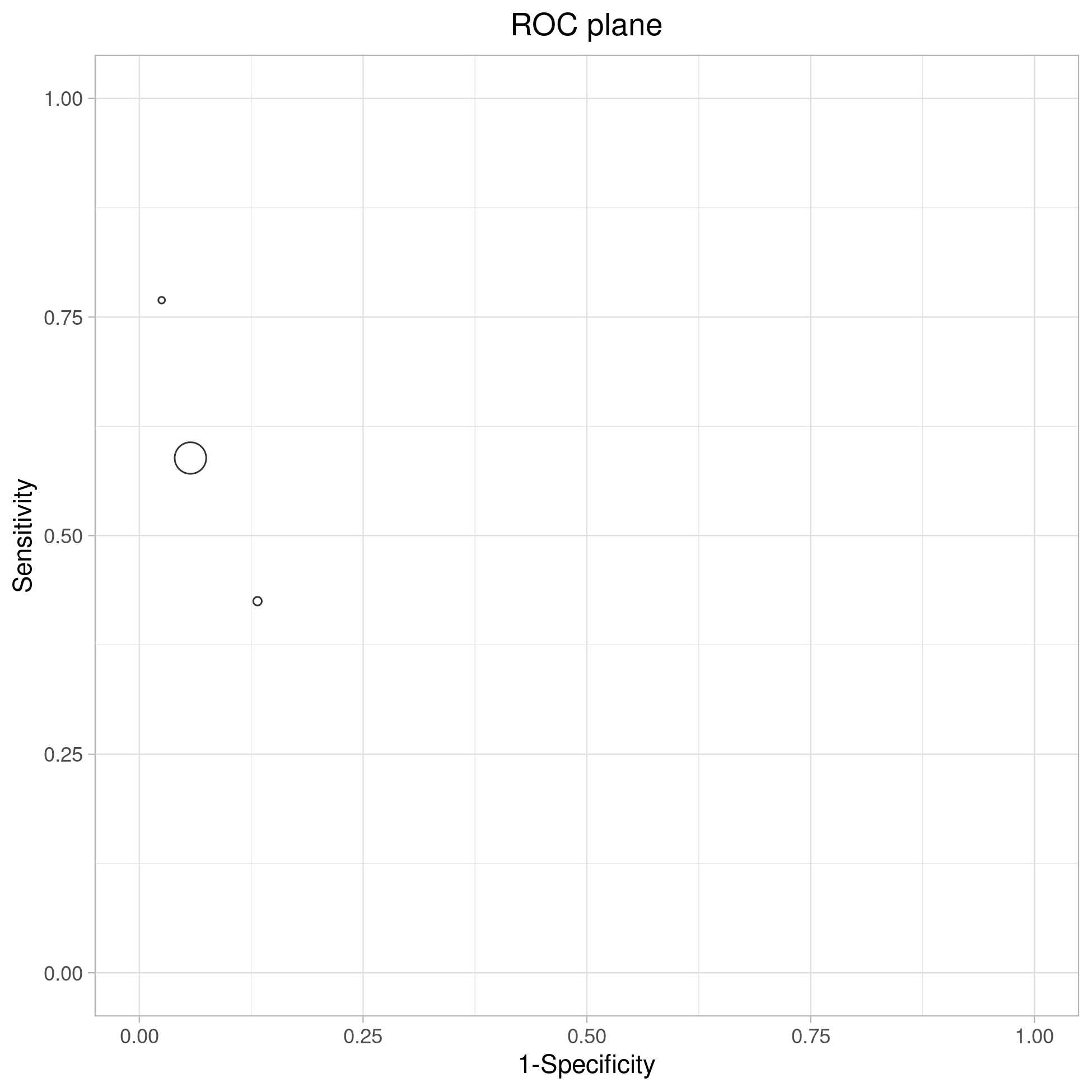

Supplement: Supplementary file 1 [file pathogens-14-00784-s001.zip › Supplementary Figure 14.png]

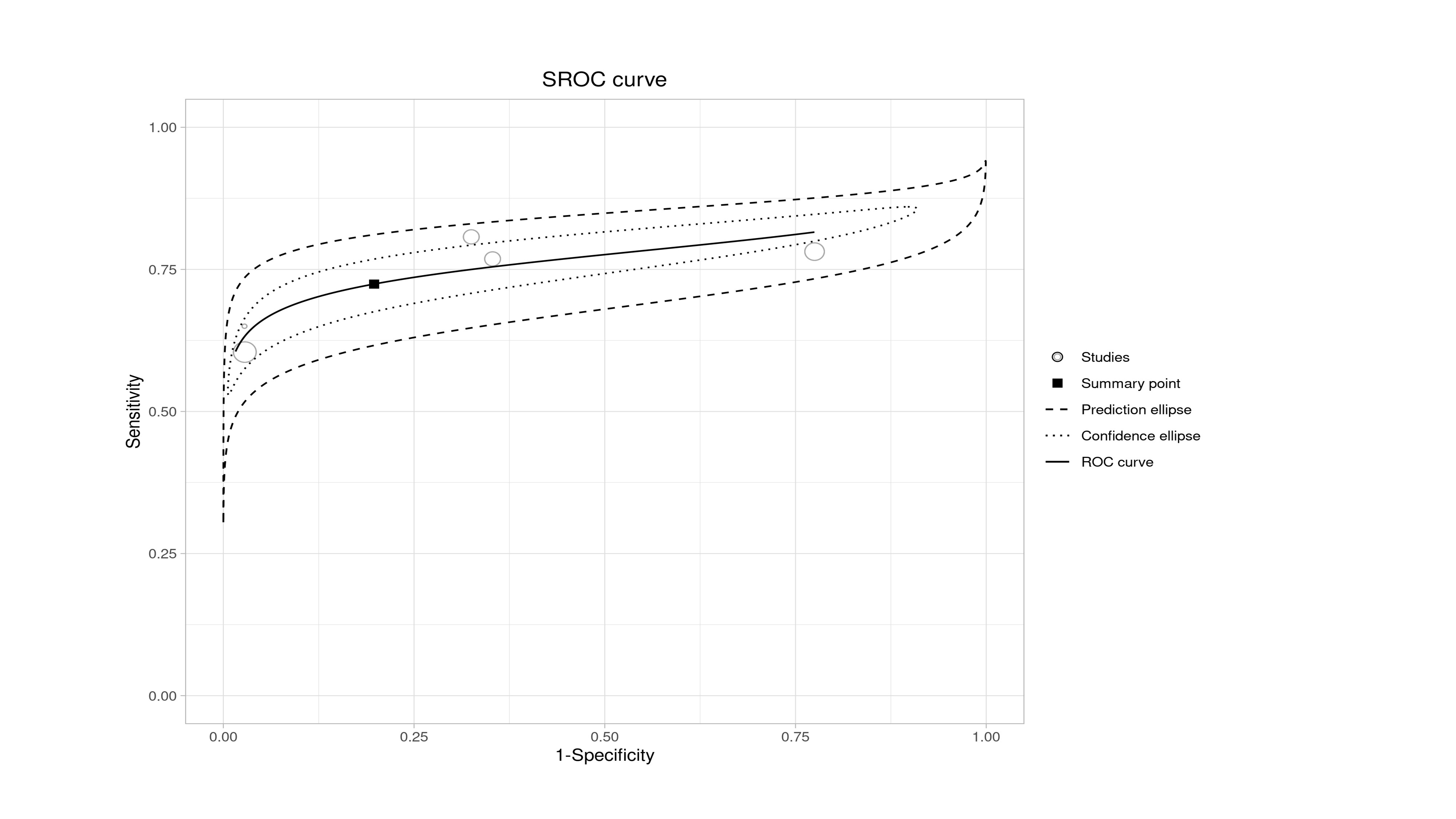

Supplement: Supplementary file 1 [file pathogens-14-00784-s001.zip › Supplementary Figure 2.jpg]

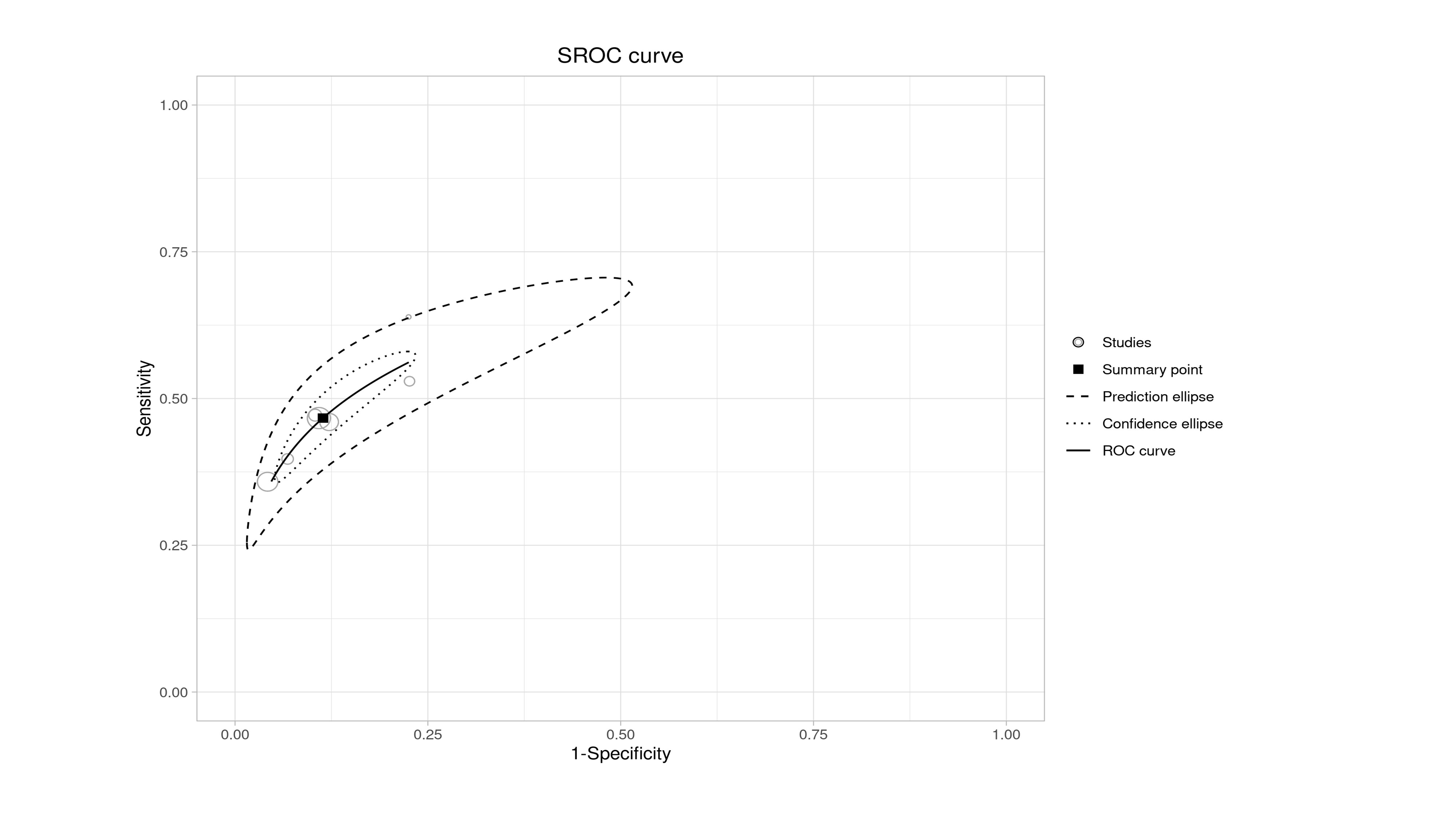

Supplement: Supplementary file 1 [file pathogens-14-00784-s001.zip › Supplementary Figure 3.jpg]

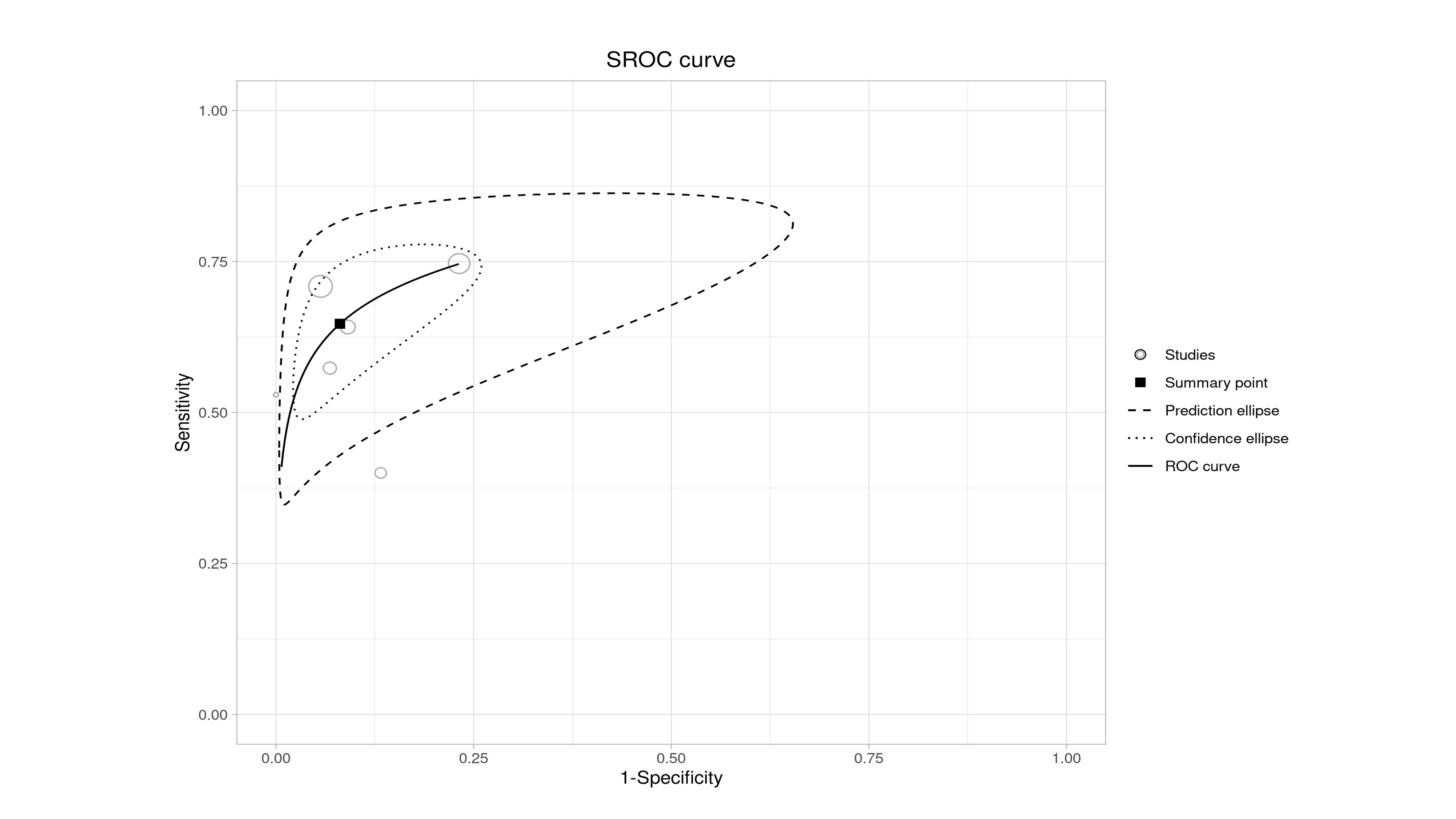

Supplement: Supplementary file 1 [file pathogens-14-00784-s001.zip › Supplementary Figure 4.jpg]

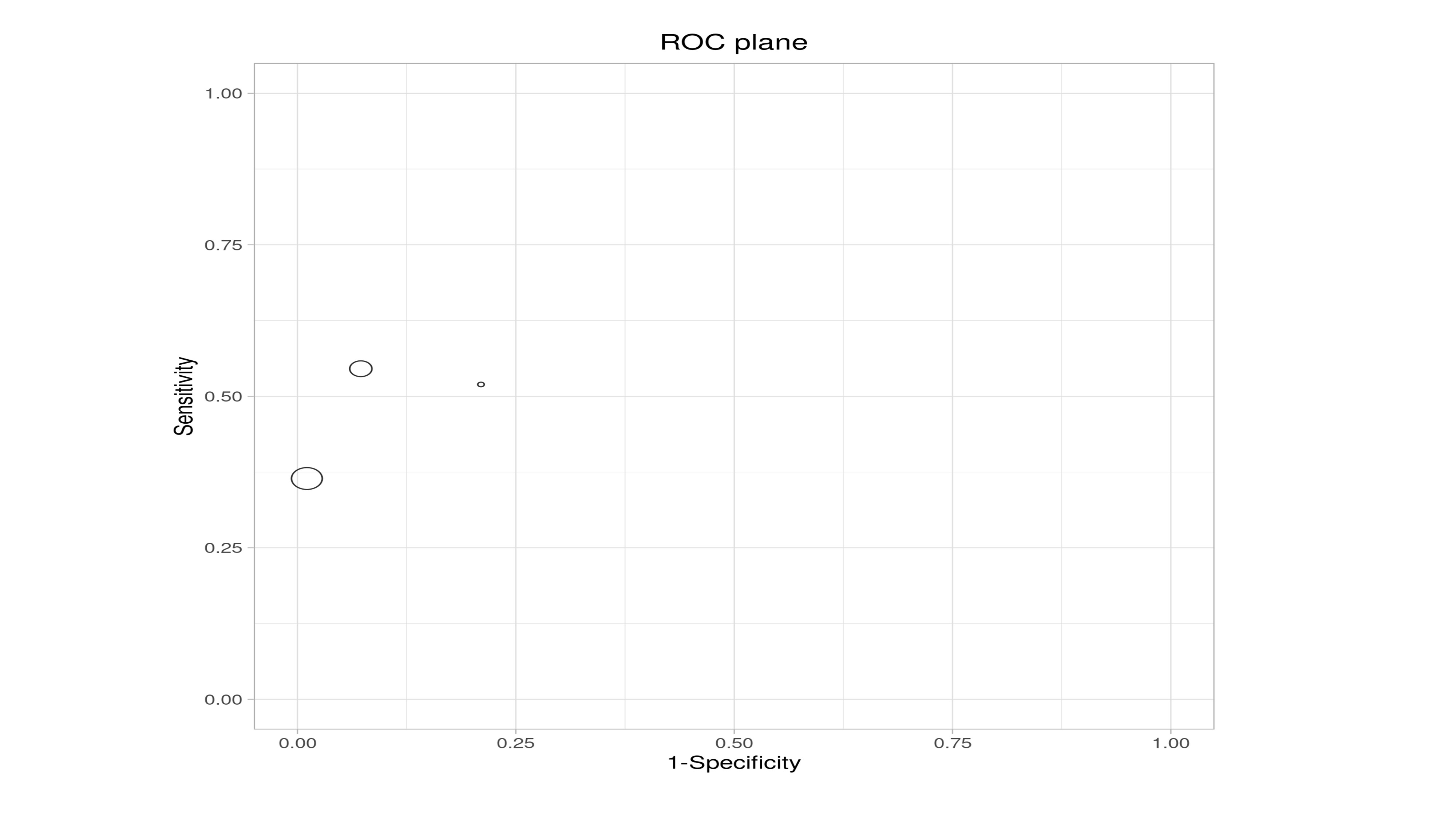

Supplement: Supplementary file 1 [file pathogens-14-00784-s001.zip › Supplementary Figure 5.jpg]

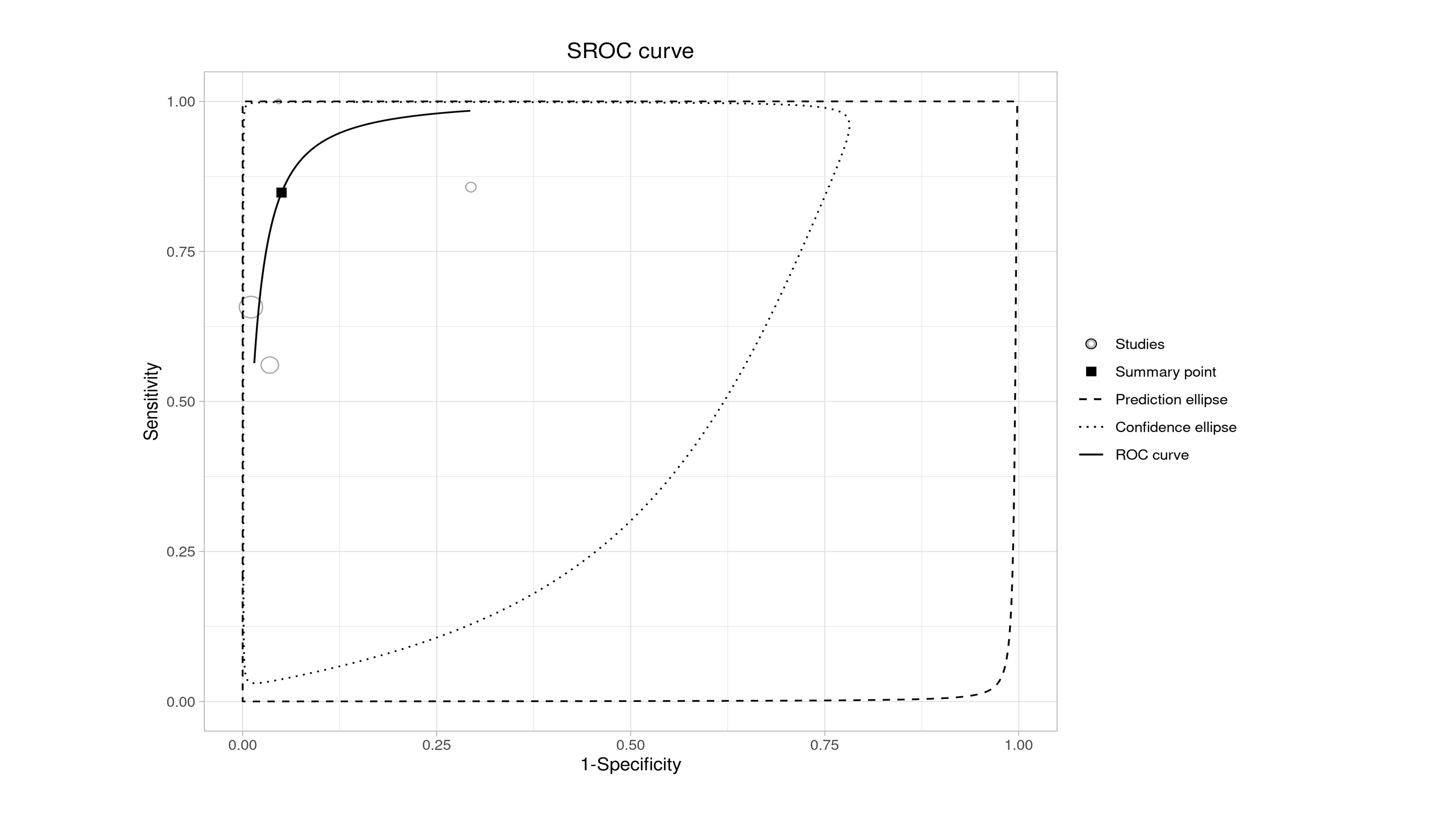

Supplement: Supplementary file 1 [file pathogens-14-00784-s001.zip › Supplementary Figure 6.jpg]

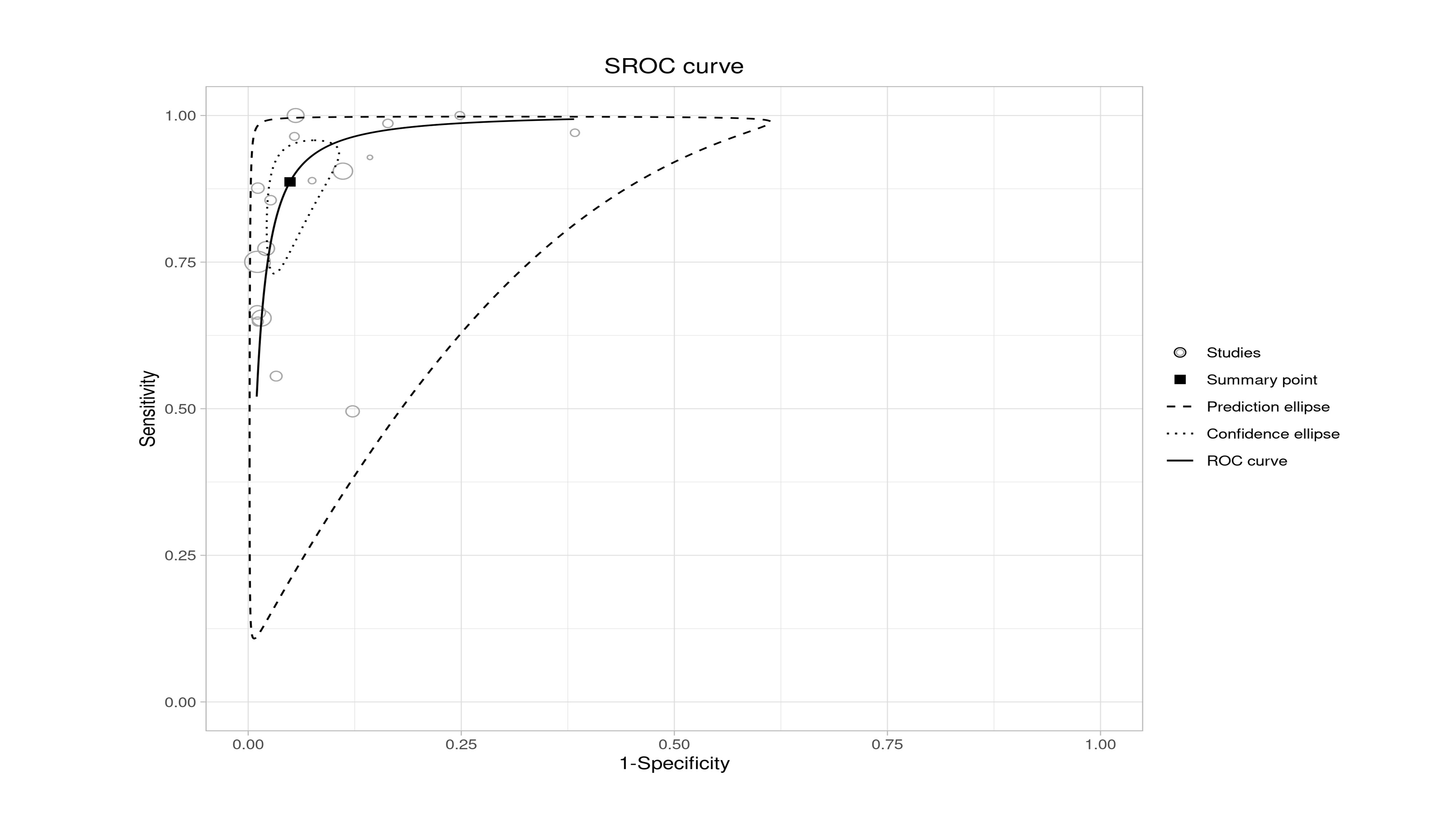

Supplement: Supplementary file 1 [file pathogens-14-00784-s001.zip › Supplementary Figure 7.jpg]

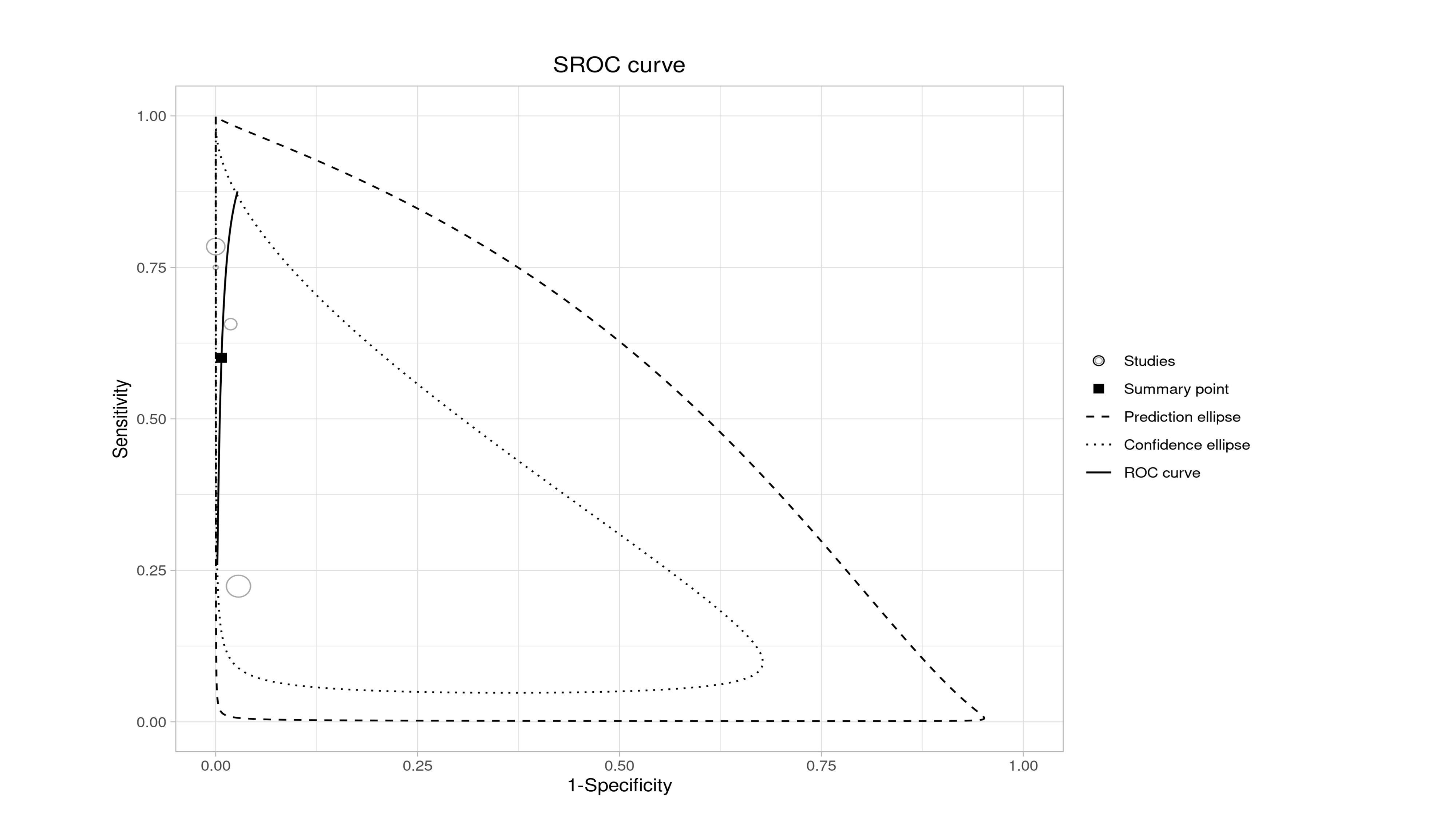

Supplement: Supplementary file 1 [file pathogens-14-00784-s001.zip › Supplementary Figure 8.jpg]

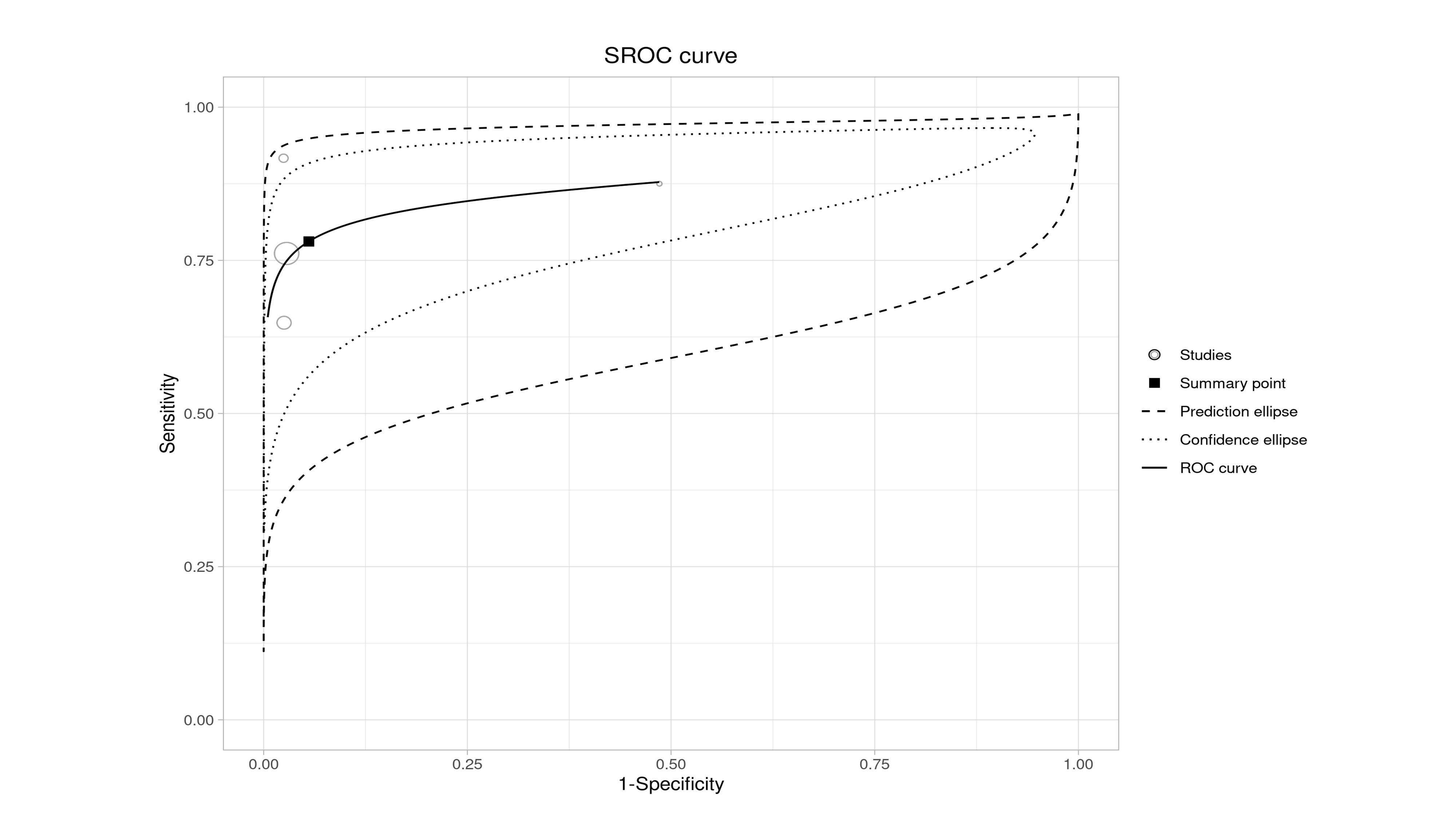

Supplement: Supplementary file 1 [file pathogens-14-00784-s001.zip › Supplementary Figure 9.jpg]
